# Supplementary material for: Global economic costs of alien birds
Source: PLoS One. 2023 Oct 18;18(10):e0292854. doi: 10.1371/journal.pone.0292854 (PMC10584179; doi:10.1371/journal.pone.0292854)
Supplement: S1 Fig — k = thousand; m = million; bn = billion. Dag = costs associated with damage to agriculture; Dfa = costs associated with damage to facilities/infrastructure/buildings; M = cost associated with management. This map was made with Natural Earth. Free vector and raster map data @ naturalearthdata.com. (DOCX) [file pone.0292854.s001.docx]

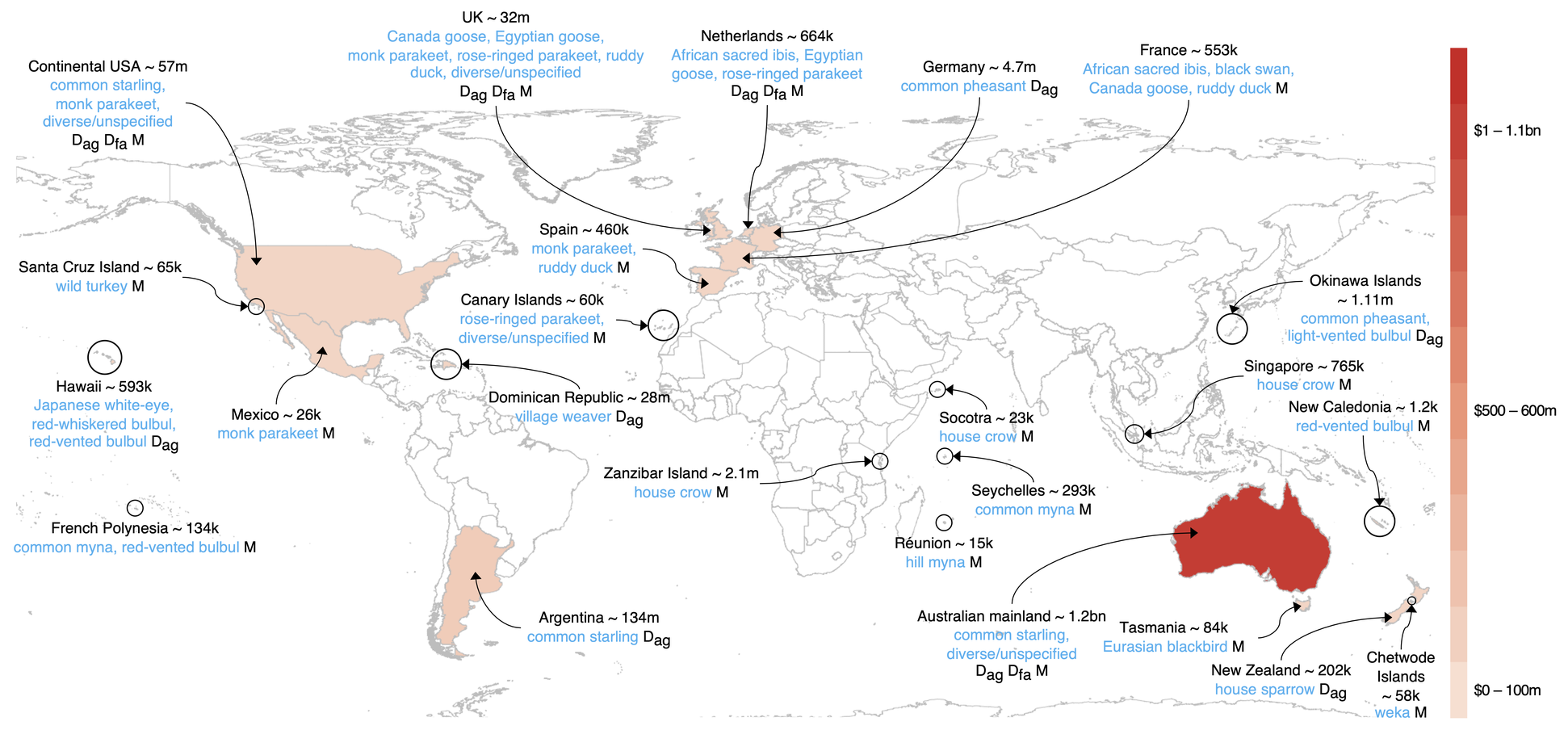


**S1 Fig. The spatial distribution of the observed economic costs associated with alien birds, with costs for street pigeons excluded (US$, 2017 exchange rate).** k = thousand; m = million; bn = billion. D_ag_ = costs associated with damage to agriculture; D_fa_ = costs associated with damage to facilities/infrastructure/buildings; M = cost associated with management. This map was made with Natural Earth. Free vector and raster map data @ naturalearthdata.com.
