# Supplementary material for: Global economic costs of alien birds
Source: PLoS One. 2023 Oct 18;18(10):e0292854. doi: 10.1371/journal.pone.0292854 (PMC10584179; doi:10.1371/journal.pone.0292854)
Supplement: S1 Table — (DOCX) [file pone.0292854.s002.docx]

**S1 Table. Alien bird species with no data on economic costs that have been assessed as having ‘harmful’ environmental impacts under the *Environmental Impact Classification for Alien Taxa* (EICAT) framework (Blackburn *et al*. 2014).**

| **Species** | **Common name** | **EICAT impact category** | **EICAT impact mechanism** | **Location of impact** |
| --- | --- | --- | --- | --- |
| *Anas platyrhynchos* | mallard | Major (MR) | Hybridisation | Lord Howe Island (Australia) |
| *Bubo virginianus* | great horned owl | Massive (MV) | Predation | Marquesas Islands (French Polynesia) |
| *Cacatua galerita* | sulphur-crested cockatoo | Moderate (MO) | Grazing / herbivory / browsing | Rock Islands (Palau) |
| *Carpodacus mexicanus* | house finch | Moderate (MO) | Disease transmission | Hawai'i (USA) |
| *Circus approximans* | swamp harrier | Major (MR) | Predation | Tahiti, Moorea (French Polynesia) |
| *Cygnus olor* | mute swan | Moderate (MO) | Grazing / herbivory / browsing | Maryland (mainland USA) |
| *Dicrurus macrocercus* | black drongo | Moderate (MO) | Predation | Northern Mariana Islands |
| *Foudia madagascariensis* | red fody | Moderate (MO) | Competition | Rodrigues Island (Mauritius) |
| *Gallus varius* | green junglefowl | Major (MR) | Competition | Cocos (Keeling) Islands (Australia) |
| *Molothrus bonariensis* | shiny cowbird | Moderate (MO) | Parasitism | Puerto Rico, Saint Lucia, Martinique |
| *Nesoenas picturata* | Madagascar turtle-dove | Massive (MV) | Hybridisation | Amirante Islands (Seychelles) |
| *Passer hispaniolensis* | Spanish sparrow | Moderate (MO) | Competition | Madeira (Portugal), Canary Islands (Spain) |
| *Pavo cristatus* | common peafowl | Moderate (MO) | Predation | Miyako Islands (Japan) |
| *Pitangus sulphuratus* | great kiskadee | Massive (MV) | Predation | Bermuda |
| *Platycercus elegans* | crimson rosella | Moderate (MO) | Competition | Norfolk Island (Australia) |
| *Psittacula alexandri* | red-breasted parakeet | Moderate (MO) | Competition | Singapore |
| *Turdus philomelos* | song thrush | Moderate (MO) | Predation | Lord Howe Island (Australia) |
| *Tyto alba* | barn owl | Massive (MV) | Competition | Lord Howe Island (Australia) |

EICAT assessment data taken from Evans *et al*. (2016) and unpublished EICAT assessments completed in 2021 by Tom Allmert and Thomas Evans.

**References**

Blackburn T.M. *et al*. (2014) A Unified Classification of Alien Species based on the magnitude of their environmental impacts. *PLoS Biology*, 12, e1001850.

Evans T *et al*. (2016) Application of the Environmental Impact Classification for Alien Taxa (EICAT) to a global assessment of alien bird impacts. *Diversity and Distributions*, 22, 919 – 931.
