# Supplementary material for: Global economic costs of alien birds
Source: PLoS One. 2023 Oct 18;18(10):e0292854. doi: 10.1371/journal.pone.0292854 (PMC10584179; doi:10.1371/journal.pone.0292854)
Supplement: Attachment — Submitted filename: ResponseToComments.docx [file pone.0292854.s003.docx]

We thank both reviewers for their comments, to which we have responded in blue text below. We have highlighted changes to the revised manuscript in yellow, and provided line number references in our responses below.

**Reviewer #1**

*In general, the introduction shows too much very specific (often local) examples and too few general assumptions in my opinion. Please increase literature search, avoid describing specific examples and increase the number of cited works (for instance, take a look at alien parakeets in Europe).*

**Response #1**: We have improved the Introduction by providing more context on alien species and invasive alien species (lines 38–42). We have also increased the number of citations, including large-scale studies that do not focus on specific species (lines 48–56). The text on these large-scale studies reads as follows:

“Other studies have reviewed or assessed the impacts of all alien bird species from an entire order of birds, such as alien Psittaciformes (parrots) in the USA and UK^7^, Europe^8^, and worldwide^9^. Another study has identified the negative impacts of alien birds caused by a specific type of impact (predation) on small islands^10^. Other studies have focussed on alien birds as an entire taxonomic class, including global reviews of their impacts^11–13^, and assessments that have quantified and categorised their environmental and socio-economic impacts by their severity and type^14,15^, and that have identified factors that make native species vulnerable to these impacts^16^.”

However, we think the specific examples give some context to the Introduction. For readers who do not understand how alien birds cause negative impacts, these examples provide solid, local examples. Based on the additional review comment below (Response #6), we have chosen to keep the example of predation by the barn owl (lines 43–45), and have added two more examples of different types of specific impacts (lines 45–48). The text now reads as follows:

“Some studies have focussed on specific alien bird species (e.g., predation of native seabirds by the barn owl (*Tyto alba*) on Hawaii^4^; hybridisation between the Chinese hwamei (*Garrulax canorus*) and the native Taiwan hwamei (*Garrulax taewanus*) in Taiwan^5^; and competition for nest-cavities between the rose-ringed parakeet (*Alexandrinus krameri*) and the native Eurasian nuthatch (*Sitta europaea*) in Belgium^6^.”

*Still I am confused: does InvaCost include also data on alien birds or, as you said at lines 75-76, alien birds are still excluded? If so, I do not understand the novelty of this work. If not, I do not understand how did you get your data. This is an important issue to be clarified, before I can provide reliable comments on the rest of the MS.*

**Response #2**: Yes, we concede that this line was unclear. *InvaCost* does include data on the costs of alien birds — this is the source of our data. We have amended the line to:

“However, data contained within the *InvaCost* database have yet to be used to assess the costs of alien birds." (lines 86–87).

We also state in the first line of the Methods that we extracted data on the economic costs of alien birds from the *InvaCost* database for our analysis (lines 117–118).

*Furthermore, I would like to suggest authors to discuss more about Figure 1, as in Europe several countries are not controlling alien birds, but they are spending for their damages, and these costs are not quantified.*

**Response #3**: This is a good point. We have added an additional line (and reference) on this in the Discussion (lines 254–257). The text reads as follows:

“However, it is also possible that high costs are not reported, even in high-income regions. For example, in Europe, data on the costs associated with alien species are not always accessible^39^. Therefore, some alien bird species with no cost data could in fact have high costs.”

*Lines 17-18. Do you refer to worldwide costs? Please clarify.*

**Response #4**: We have clarified this by stating now that our analysis was of ‘global cost data’ (line 19).

*Keywords. You never mentioned Columba livia as an invasive species in the abstract. Please clarify or try to find different keywords.*

**Response #5**: We have removed *Columba livia* from the list of keywords (replaced with ‘avian ecology’).

*Lines 42-43. This is a very specific example. It could be ok, but you have to look at different examples also, as being in the introductive part.*

**Response #6**: Agreed – we have added two more specific examples here (lines 45–48):

“Some studies have focussed on specific alien bird species (e.g., predation of native seabirds by the barn owl (*Tyto alba*) on Hawaii^4^; hybridisation between the Chinese hwamei (*Garrulax canorus*) and the native Taiwan hwamei (*Garrulax taewanus*) in Taiwan^5^; and competition for nest-cavities between the rose-ringed parakeet (*Alexandrinus krameri*) and the native Eurasian nuthatch (*Sitta europaea*) in Belgium^6^.”

As discussed above, we have also described large-scale studies that do not focus on specific species (lines 48–56). The text on these large-scale studies reads as follows:

“Other studies have reviewed or assessed the impacts of all alien bird species from an entire order of birds, such as alien Psittaciformes (parrots) in the USA and UK^7^, Europe^8^, and worldwide^9^. Another study has identified the negative impacts of alien birds caused by a specific type of impact (predation) on small islands^10^. Other studies have focussed on alien birds as an entire taxonomic class, including global reviews of their impacts^11–13^, and assessments that have quantified and categorised their environmental and socio-economic impacts by their severity and type^14,15^, and that have identified factors that make native species vulnerable to these impacts^16^.”

*Line 46. Please, change “demonstrates” with “shows”. You have no experiment in your manuscript.*

**Response #7**: We have changed ‘demonstrates’ to ‘shows’.

*Figure 2. Please, scientific names should be in italics.*

**Response #8**: We have amended the figure – all scientific names are now in italics.

**Reviewer #2**

*1 In the regions with reported data, it will be helpful to run a set of regression analysis to see how regions, species, taxonomic groups, impact type (environmental impact data from Reference 20), or year fixed effect can explain the variation of the economic costs. This provides stronger tests of the 6 hypotheses.*

**Response #9**: We agree with this suggestion. However, we did not aim to identify drivers of costs with this paper. Rather we aimed to undertake the first global synthesis of these costs, and to identify patterns in their spatial, temporal and taxonomic distribution. We have submitted a full, separate study to *People and Nature*, which has just been accepted for publication. That study attempts to identify links between specific types of biodiversity impacts and high costs, and factors that cause alien birds to have both severe biodiversity impacts and high costs. Together, these two complementary studies help improve our knowledge of the effects of bird invasions.

We therefore conclude that this study is best presented in its current format. However, we have included additional text at the end of the conclusion to describe some possible directions for future research (including some suggested by the reviewer) that have been informed by our study (lines 444–450):

“Indeed, our study demonstrates that we have much to learn about the economic costs of alien birds. Avenues for future research may include identifying characteristics of alien bird species that cause them to have high economic costs, and factors that cause variation in the distribution of costs across different regions. This research may prove useful in predicting the types of species that have the most damaging economic impacts, which may inform biosecurity measures to prevent their introduction as aliens.”

*2 Find some proxy of the economic cost especially in the unreported regions. The paper states that economics costs are likely to be underreported because:*

*1) Due to budget constraints, there is no monitoring/research in some regions, particularly low-income regions.*

*2) Within the reported regions, only costs associated with the most damaging species are reported. The other species may lead to relatively low costs.*

*It may be possible to use the data from the reported region to infer information about economic costs in the unreported regions. For example, if we know the reason for the invasive species and if one species appears in both reported region(s) and unreported region, can we assume the economics costs in the unreported region is proportional to the economics costs in the reported regions? The proportion can be a function of regional GDP, year, etc.*

**Response #10**: We agree – and this is also a study that we are doing, but separately. This is because there are actually relatively few bird species with cost data and because such extrapolation requires several additional methodological steps than suggested above. So we combined these data with that for other tetrapod species (to analyse costs for alien tetrapod species as a group) and conducted complex extrapolation analyses to infer the likely costs of invasive alien species without documented costs.

*3 The paper also states that some impacts are hard to monetize. So, no cost data even when the environmental impact is reported high.*

*It can also be helpful to construct a model to predict economic cost based on environmental and social impacts and regional characteristics. It would be interesting to explain the gap between the predicted economic cost and reported cost.*

**Response #11**: We entirely agree with the importance and interest of these questions, but this is a rather complex and complete study that requires to be described and presented into its own manuscript. As mentioned in Response #9, we have submitted a full, separate study to *People and Nature*, which has just been accepted for publication. That study attempts to identify links between specific types of biodiversity impacts and high costs, and factors that cause alien birds to have both severe biodiversity impacts and high costs. Together, the current manuscript and the accepted manuscript help improve our knowledge of the effects of bird invasions.
