# Supplementary material for: Global economic costs of alien birds
Source: PLoS One. 2023 Oct 18;18(10):e0292854. doi: 10.1371/journal.pone.0292854 (PMC10584179; doi:10.1371/journal.pone.0292854)
Supplement: Attachment — Submitted filename: ResponseToComments240823.docx [file pone.0292854.s004.docx]

We thank the reviewer for these additional comments, which we have addressed below in blue text. We have highlighted changes to the revised manuscript in yellow, and provided line number references in our responses below.

**Reviewer #2**

*The paper investigates the distribution of economic costs arising from alien birds, focusing on spatial, temporal, and species variations. The primary approach involves a descriptive analysis of the cost distribution and underlying mechanisms. While the research provides valuable insights, there are some aspects that require clarification and additional support to strengthen the overall findings.

1 Support for Hypothesis 3 (more cost data are available for damaging alien bird species because they are more likely to be managed.)

While the paper mentions that 60% of alien bird species with costs have harmful environmental impacts, it would be more informative to provide a summary of the percentage of each environmental impact category under the Environmental Impact Classification for Alien Taxa (EICAT) framework) to understand the severity of their effects accurately.*

**Response #1:** Hypothesis 3 states that more cost data will be available for alien bird species with damaging environmental impacts because they are more likely to be managed (lines 107-110).

We have amended Figure 3 (Figure 2 in the previously submitted version of the manuscript) by adding the most severe reported impact caused by each alien bird species, as assessed using the *Environmental Impact Classification for Alien Taxa* (EICAT) framework. Figure 3 now shows that 60% of the species with costs have impacts considered to be ‘harmful’ under EICAT (i.e., MO, MR, or MV impacts). We refer to Figure 3 at line 419.

*2 Support for Hypothesis 4 (data on costs will be unavailable for some regions where severe environmental impacts are reported as environmental impacts are hard to monetize.)

The evidence supporting Hypothesis 4, which suggests that data on costs may be unavailable in regions with severe environmental impacts, lacks substantial support from the data. Calculating the percentage of alien bird species with damaging costs among those with "harmful" or higher impacts would offer more compelling evidence for this hypothesis.*

**Response #2:** Hypothesis 4 states that the biodiversity impacts of alien birds are sometimes difficult to monetise and therefore there will be regions with severe biodiversity impacts, but no cost data (lines 110-114).

This is a good point. We have calculated the percentage of alien bird species with ‘harmful’ biodiversity impacts (as assessed using EICAT) that do not have reported costs (58%) (lines 390 – 406).

*3 Conflicting Evidence for Hypothesis 5

The paper introduces Hypothesis 5, stating that management costs for eradicating alien birds are typically low compared to their damage costs. However, there is conflicting evidence presented in the figures (Figure S4 and Figure S2). In Figure S4, damage cost dominates management costs (with and without street pigeons). But in Figure S2, you have higher percentage of management costs than damage costs when street pigeons are included. Moreover, it would be helpful if you refer to the figure that support your hypothesis.*

**Response #3:** We have given more thought to these calculations. Because costs for street pigeons were of ‘low’ reliability, and information on their specific nature was not available, we decided it was appropriate to categorise them as being ‘mixed’ costs. We have explained this in the Methods (lines 165 – 170). This means Figure S2 and Figure S4 have changed (and they are no longer conflicting). These figures are now in the main manuscript (not the supporting information). They are Figure 2 (formerly Figure S2) and Figure 5 (formerly Figure S4).

*4 Statement in the abstract and Support for Hypothesis 6.*

*In the Abstract (Line 24), the paper states that "Research to understand why (alien birds tend to have lower costs than many other alien taxa (Hypothesis 6)) may inform measures to reduce costs associated with biological invasions".

With limited and potentially underreported data, it is hard to justify hypothesis 6.* *Moreover, the paper discusses the two following reasons of why alien birds have lower costs than the other taxa, but there is no concrete evidence to support these mechanisms:

a). Lower costs for alien birds may come from underestimation:

The paper acknowledges that costs for alien birds may be underestimated due to underreported environmental costs that are challenging to monetize. Additionally, minor environmental and socio-economic impacts of alien birds have led to limited research. These points provide valuable insights into potential reasons for the lower perceived costs of alien birds. However, it would be beneficial to include specific examples or case studies to illustrate the extent of underestimation and its impact on cost assessments.*

**Response #4:** There are two points in this comment: (*i*) that biodiversity impacts of alien birds are difficult to monetise, and (*ii*) that minor impacts (biodiversity or socio-economic impacts) of alien birds do not attract research (and therefore, we do not have impact data for many species, probably because their impacts are minor).

(*i*) We have included a table in the Supporting Information (Table S1) that lists alien bird species with ‘harmful’ impacts (as assessed using EICAT) for which no cost data are available. We refer to Table S1 at line 406. This lends weight to our argument that the biodiversity impacts of alien birds can be difficult to monetise.

(*ii*) We have included an example of an alien bird species that has been present in New Zealand for over 150 years (and is now widespread in southern regions), but that is *Data Deficient* under EICAT because there are no data quantifying its impacts (most likely because these impacts are minor) (lines 338 – 344). We also refer to previous studies that have assessed the environmental impacts, socio-economic impacts, and monetary costs of alien birds, and concluded that these impacts are likely minor for many species, and this is why they are Data Deficient for impacts and costs (lines 346 – 348).

Furthermore, there is no evidence that birds are less well studied than other taxonomic groups of alien species. On the contrary, they are well studied compared to many other taxonomic groups. There is therefore no reason to think that research bias could be responsible for alien birds having low costs compared to other taxonomic groups of alien species.

*b). Mechanisms for Lower Costs in Alien Birds than other taxa:

The paper suggests possible mechanisms that could explain why alien birds tend to have lower costs compared to other taxa, such as their habitat preference (above ground or water level). Nevertheless, the paper lacks concrete evidence to support these mechanisms. For example, there is no information provided to demonstrate whether the environmental impacts of alien birds are indeed smaller than those of ants and crayfish. Low environmental costs of alien birds might lead to low costs. However, together with costs underestimation, high environmental impacts of alien birds may end up with lower costs reflected by data. Including empirical data or relevant studies to validate these hypotheses would strengthen the paper's argument.*

**Response #5:** We suggested that the characteristics of birds might make them easier to identify, contain, remove, or eradicate compared to other taxonomic groups of alien species such as ants, crayfish, and bivalves. This is because they are more conspicuous than many invertebrate species, and do not attach themselves to structures or live below water like crayfish and bivalves, for example (lines 370 – 383).

In response to the need to include empirical data to strengthen this argument, we have added an example of the difficulty in detecting ants in Australia, which has resulted in extensive survey work (lines 374 – 377), and an example of the huge costs caused by alien bivalves in the USA due to their ability to attach themselves to structures (lines 379 – 381).

Underestimation of costs is a problem for alien species in general, not just alien birds. So, we think it is reasonable to compare the total costs of different taxonomic groups of alien species.

*5 Is Inva cost the only dataset that provides global information about cost of alien birds? Is there any other dataset available even locally? Other relevant data will be able to support the arguments.*

**Response #6:** *InvaCost* includes data on alien birds from all sources, including local sources where available. It is therefore the most comprehensive database on the economic costs of alien species (Ahmed et al. 2023. *BioScience* doi:10.1093/biosci/biad060).

*Overall, the paper provides valuable insights into the distribution of economic costs associated with alien birds, considering spatial, temporal, and species-related variations.*

Thank you.

*However, the descriptive nature of the data analysis limits the paper's ability to draw robust conclusions. By incorporating specific examples, empirical data, and further support for the hypothesis and proposed mechanisms, the authors can enhance the paper's impact and provide more practical implications for managing biological invasions linked to alien bird species.*

We appreciate these comments and suggestions, and we hope that our revisions have adequately addressed them.
