# Supplementary material for: Global economic costs of alien birds
Source: PLoS One. 2023 Oct 18;18(10):e0292854. doi: 10.1371/journal.pone.0292854 (PMC10584179; doi:10.1371/journal.pone.0292854)
Supplement: Attachment — Submitted filename: Response_to_comments200923.docx [file pone.0292854.s005.docx]

We thank the reviewer for raising this additional point, which we have addressed below in blue text. We have highlighted changes to the revised manuscript in yellow, and provided line number references in our responses below.

**Reviewer #2**

*Thank you for addressing my comments. I only have 1 question now.

1 Reliability of Street Pigeon Cost Data:
1) Dataset Information (lines 136-137):

The dataset offers descriptions of the reliability for cost records, categorizing them as 'low' or 'high'.

2) Reference from lines 172-173:

A significant portion (95%) of the cost records (n=333) are marked as 'high' reliability. It's unclear if this percentage includes the costs associated with street pigeons.

3) Reference from lines 163-164:

The costs related to street pigeons have been designated a 'low' reliability and are categorized under 'mixed' costs.

Concerns:
There is ambiguity about the reliability classification for street pigeon costs in the dataset, especially since they constitute over half of all costs (US $2 billion out of US $3.6 billion from reference line 169-171).

If the dataset classifies street pigeon costs as 'high reliability' but they are later marked as 'low', this discrepancy could affect the reliability of the overall cost data (US$3.6 billion). This is particularly important considering:*

*a) Street pigeons contribute to 50% of the total costs (US $2 billion).
b) The validity of the 'high' reliability label for other birds might be brought into question.

Recommendations:

Kindly provide clarity on the reliability rating assigned to street pigeon costs in the dataset.*

**Response #1:** We acknowledge that the information in the Results was perhaps less clear than we had intended regarding the reliability category for street pigeon costs. They were allocated to the ‘low’ reliability category, and we have made this clear in the revised manuscript (lines 169 – 171).

*It would also be beneficial to include an explanatory note for the "mixed" category (representing both management and damage) in relevant figures or tables.*

**Response #2:** We have added text to the legend for Figure 2 and Figure 5 to describe mixed costs, and to note that all street pigeon costs were allocated to this category.
